# Prediction Pathway for Severe Asthma Exacerbations: A Bayesian Network Analysis

**DOI:** 10.1016/j.chest.2025.04.046

**Published:** 2025-05-19

**Authors:** Chandra Prakash Yadav, Atlanta Chakraborty, David B. Price, Laura Huey Mien Lim, Yah Ru Juang, Richard Beasley, Mohsen Sadatsafavi, Christer Janson, Mariko Koh Siyue, Eileen Wang, Michael E. Wechsler, David J. Jackson, John Busby, Liam G. Heaney, Paul E. Pfeffer, Bassam Mahboub, Diahn-Warng Perng, Borja G. Cosio, Luis Perez-de-Llano, Riyad Al-Lehebi, Désirée Larenas-Linnemann, Mona S. Al-Ahmad, Chin Kook Rhee, Takashi Iwanaga, Enrico Heffler, Giorgio Walter Canonica, Richard W. Costello, Nikolaos G. Papadopoulos, Andriana I. Papaioannou, Celeste M. Porsbjerg, Carlos A. Torres-Duque, George C. Christoff, Todor A. Popov, Mark Hew, Matthew J. Peters, Peter G. Gibson, Jorge Máspero, Celine Bergeron, Saraid Cerda, Elvia Angelica Contreras, Wenjia Chen

**Affiliations:** a Saw Swee Hock School of Public Health, National University of Singapore, Ann Arbor, MI; b University of Michigan, Ann Arbor, MI; c Optimum Patient Care Global, Cambridge, England; d Observational and Pragmatic Research Institute, Singapore; e Division of Applied Health Sciences, Centre of Academic Primary Care, University of Aberdeen, Aberdeen, Scotland; f Medical Research Institute of New Zealand, Wellington, New Zealand; g Respiratory Evaluation Sciences Program, Faculty of Pharmaceutical Sciences, University of British Columbia, Vancouver, BC, Canada; h Department of Medical Sciences, Uppsala University, Uppsala, Sweden; i Duke-NUS Medical School, Singapore; j Department of Respiratory and Critical Care Medicine, Singapore General Hospital, Singapore; k Division of Allergy and Clinical Immunology, Department of Medicine, National Jewish Health, Denver, CO; l Division of Allergy and Clinical Immunology, Department of Medicine, University of Colorado School of Medicine, Aurora, CO; m NJH Cohen Family Asthma Institute, Department of Medicine, National Jewish Health, Denver, CO; n UK Severe Asthma Network and National Registry, Guy’s and St Thomas’ NHS Trust, London, England; o School of Immunology and Microbial Sciences, King’s College London, London, England; p Centre for Public Health, Queen’s University Belfast, Belfast, Northern Ireland; q Wellcome-Wolfson Centre for Experimental Medicine, Queen’s University Belfast, Belfast, Northern Ireland; r Department of Respiratory Medicine, Barts Health NHS Trust, London, England; s Barts and the London School of Medicine and Dentistry, Queen Mary University of London, London, England; t College of Medicine, University of Sharjah, Sharjah, United Arab Emirates; u Rashid Hospital, Dubai Health Authority, Dubai, United Arab Emirates; v Division of Clinical Respiratory Physiology, Chest Department, Taipei Veterans General Hospital, Taipei City, Taiwan; w COPD Assembly of the Asian Pacific Society of Respirology, Tokyo, Japan; x Son Espases University Hospital-IdISBa-CIBERES, Mallorca, Spain; y Pneumology Service, Lucus Augusti University Hospital, EOXI Lugo-Monforte-Cervo, Lugo, Spain; z Biodiscovery Research Group, Health Research Institute of Santiago de Compostela, Santiago de Compostela, Spain; aa Department of Pulmonology, King Fahad Medical City, Riyadh, Saudi Arabia; bb College of Medicine, Alfaisal University, Riyadh, Saudi Arabia; cc Centro de Excelencia en Asma y Alergia, Hospital Médica Sur, Mexico City, Mexico; dd Microbiology Department, College of Medicine, Kuwait University, and Al-Rashed Allergy Center, Ministry of Health, Kuwait City, Kuwait; ee Division of Pulmonary and Critical Care Medicine, Department of Internal Medicine, Seoul St. Mary’s Hospital, and College of Medicine, Catholic University of Korea, Seoul, South Korea; ff Sleep Medicine Center, Kindai University Hospital, Osakasayama, Japan; gg Personalized Medicine, Asthma and Allergy, Humanitas Clinical and Research Center, IRCCS, Milan, Italy; hh Department of Biomedical Sciences, Humanitas University, Pieve Emanuele, Milan, Italy; ii Clinical Research Centre, Beaumont Hospital, and Department of Respiratory Medicine, RCSI, Dublin, Ireland; jj Division of Infection, Immunity and Respiratory Medicine, University of Manchester, Manchester, England; kk Allergy Department, 2nd Pediatric Clinic, University of Athens, Athens, Greece; ll 2nd Respiratory Medicine Department, National and Kapodistrian University of Athens Medical School, and Attikon University Hospital, Athens, Greece; mm Respiratory Research Unit, Bispebjerg University Hospital, Copenhagen, Denmark; nn CINEUMO, Respiratory Research Center, Fundación Neumológica Colombiana, Bogotá, Colombia; oo Medical University-Sofia, Faculty of Public Health, Sofia, Bulgaria; pp Clinic of Occupational Diseases, University Hospital “Sv. Ivan Rilski,” Sofia, Bulgaria; qq Allergy, Asthma and Clinical Immunology Service, Alfred Health, Melbourne, Australia; rr Public Health and Preventive Medicine, Monash University, Melbourne, Australia; ss Department of Thoracic Medicine, Concord Hospital, Sydney, Australia; tt Australian Severe Asthma Network, Priority Research Centre for Healthy Lungs, University of Newcastle, Newcastle, Australia; uu Hunter Medical Research Institute, Department of Respiratory and Sleep Medicine, John Hunter Hospital, New Lambton Heights, Australia; vv Clinical Research for Allergy and Respiratory Medicine, CIDEA Foundation, Buenos Aires, Argentina; ww University Career of Specialists in Allergy and Clinical Immunology, Buenos Aires University School of Medicine, Buenos Aires, Argentina; xx Centre for Lung Health, Vancouver General Hospital, University of British Columbia, Vancouver, BC, Canada; yy Medical Specialties Unit, Secretary of National Defense, Mexico City, Mexico; zz Mexican Council of Clinical Immunology and Allergy, Mexico City Office, Mexico City, Mexico; aaa Department of Allergy and Clinical Immunology, Lic. Adolfo López Mateos Regional Hospital of the Institute of Security and Social Services for State Workers (ISSSTE), Mexico City, Mexico

**Keywords:** asthma, Bayesian network, causal prediction, influence diagram, model validation, risk prediction, severe exacerbation

## Abstract

**Background:**

Accurate risk prediction of exacerbations is pivotal in severe asthma management. Multiple risk factors are at play, but the pathway of risk prediction remains unclear.

**Research Question:**

How do the interplays of clinically relevant predictors lead to severe exacerbations in patients with severe asthma?

**Study Design and Methods:**

Patients with severe asthma (n = 6,814, aged ≥ 18 years), biologic naive, were identified from the Severe Asthma Registry (2017-2021). Relevant predictors covered demographics, lung function, inflammation biomarkers, health care use, medications, exacerbation history, and comorbidities. A Bayesian network, representing the prediction process of severe exacerbations, was obtained by combining expert knowledge and machine learning algorithms. Internal validation was performed. The proposed influence diagram integrated decision and utility nodes into the prediction pathway.

**Results:**

The Bayesian network analysis revealed that blood eosinophil count, fractional exhaled nitric oxide level, and FEV_1_ directly influenced the transition between prior and future severe exacerbations. The presence of chronic rhinosinusitis indirectly affected such transition by directly influencing blood eosinophil count, fractional exhaled nitric oxide, and % predicted FEV_1_. Macrolide use independently affected history of exacerbations to influence future severe asthma exacerbations. Model discrimination was moderate in 10-fold cross-validation and leave-1-country-out cross-validation, and model calibration was high in train-test data.

**Interpretation:**

This study identified an essential prediction pathway of severe exacerbation, which involves the influence of chronic rhinosinusitis on the immediate predictors of risk transition from current to future severe asthma exacerbations. Macrolide use was another essential prediction pathway identified. The findings support shared clinical decision-making in severe asthma treatment.

FOR EDITORIAL COMMENT, SEE PAGE 277

**Take-Home Points**

**Study Question:** How do clinically relevant predictors interact in leading to severe exacerbations in patients with severe asthma?

**Results:** Blood eosinophil count, fractional exhaled nitric oxide (Feno) level, and % predicted FEV_1_ directly influenced the transition between prior and future severe exacerbations, whereas the presence of chronic rhinosinusitis (CRS) indirectly affected such transition by directly influencing blood eosinophil count, Feno, and % predicted FEV_1_.

**Interpretation:** This study identified an essential prediction pathway of severe exacerbation, which involves the influence of CRS on the immediate predictors of risk transition from current to future severe asthma exacerbations.

People with severe asthma suffer from ongoing risk of exacerbations and impaired lung function,^1^^,^^2^ with approximately 20% dependent on oral corticosteroids (OCSs) for flare-up relief and maintenance.^3^ Guideline-based management of severe asthma focuses on reducing severe exacerbations requiring OCS and/or acute care.^4^ Accurate identification of patients with severe asthma with a high risk of severe exacerbations enables efficient preventive strategies and treatment escalation, and empowers shared decision-making.^5^ Well-established predictors of asthma exacerbations include prior history of exacerbations, asthma-related ED visits and hospitalizations, poor asthma control, biomarkers including blood eosinophil counts (BECs),^6^ fractional exhaled nitric oxide (Feno),^7^ OCS use, and rescue medications, as well as lung function,^8^, ^9^, ^10^ based on which several asthma risk prediction models have been developed. For instance, Couillard and colleagues^11^^,^^12^ developed a prototype Oxford Asthma Attack Risk Scale (ORACLE) scale, which quantifies the excess risk of asthma exacerbations based on BEC and Feno while considering exacerbation history. Meanwhile, the proliferation of large-scale electronic health records has advanced the development of machine learning (ML)-based asthma prediction models.^13^ However, most of those current models are limited by applicability and generalizability, as the simpler models have inadequate prediction performance^10^ while the more advanced models are relatively complex and lack transparency.^14^

Besides, to further understand how the predictors interact in leading to an asthma exacerbation, it is imperative to delineate their interactions to uncover their specific roles in the core prediction pathway. The incorporation of core predictor interplay can enhance prediction accuracy, and a transparent representation of the prediction process can reinforce physician trust and inform modifiable steps and consequences in clinical decision-making. However, a lack of such information to date presents a knowledge gap.

In this regard, Bayesian network (BN) analysis is a powerful tool to address these gaps. The BNs depict conditional independence among predictors graphically, with nodes (predictors) and edges (probabilistic relationships). By combining expert knowledge with ML, BNs can identify probabilistic relationships among predictors, allowing flexible modeling of complex relationships and the capturing of associative links, which, combined with expert opinion, may infer causality.^15^^,^^16^ The BN enables knowledge translation of clinical risk prediction and facilitates counterfactual prediction analysis.^17^

BN analysis has been previously applied to epidemiologic and predictive modeling, including the detection of relations between genes, environment, and disease,^18^ as well as the identification of clinical markers of COPD exacerbation risk.^19^ However, there was no such analysis to understand the interactive pathways of risk factors leading to adverse health outcomes in patients with asthma.

Using data from the International Severe Asthma Registry (ISAR),^20^ with more than 12,000 patients with severe asthma from 20 countries (2017-2021), this study aimed to construct a BN representing the associative interaction network among key predictors leading to severe exacerbations in patients with severe asthma and to develop an influence diagram^21^ to support clinical decision-making in severe asthma management.

## Study Design and Methods

### Data Source

The data were retrieved from the ISAR, comprising 12,458 patients with severe asthma,^20^ during 2017-2021. The ISAR collected individual-level registry data mainly from asthma specialist clinics, including patient demographics, lifestyle factors, clinical features, lung function, biomarkers, comorbidities, health service use, medication use, and outcomes including severe asthma exacerbation.

### Index Date

The index date of a patient was defined as the date of enrollment in the asthma specialist clinic to join the ISAR study.

### Study Sample

We identified patients who were aged ≥ 18 years and with a diagnosis of severe asthma, defined as receiving Global Initiative for Asthma (GINA) step 5 treatment or with severe asthma remaining uncontrolled at GINA step 4 at baseline visit. Definitions of diagnosis were generally consistent with the European Respiratory Society and American Thoracic Society definitions of severe asthma^22^ (e-Appendix 1, e-Table 1).

**Figure 1:**
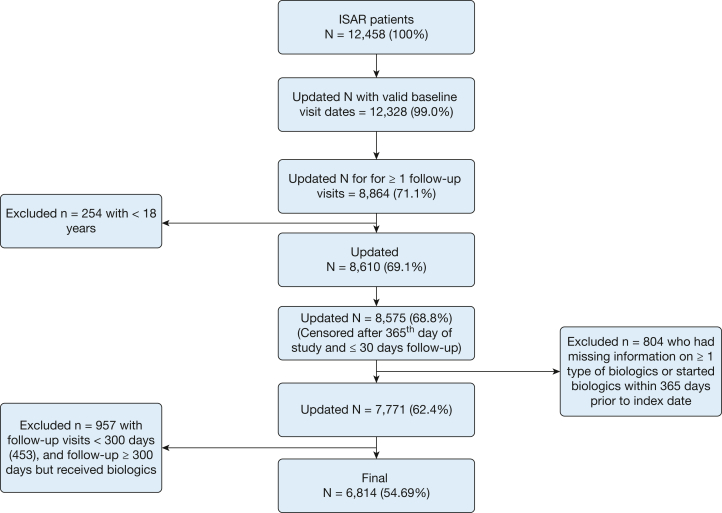
Patient flow diagram of the International Severe Asthma Registry (ISAR) cohort.

Patients who were currently receiving any biologic therapy were excluded from the analysis. The follow-up period was limited to a maximum of 365 days. Individuals with fewer than 300 days of follow-up from the index date were excluded to ensure adequate outcome measurement, as the analysis did not incorporate follow-up time as a predictor or offset variable. As a result, 6,814 individuals (55%) met these criteria. Patient flow is outlined in Figure 1. The characteristics of study participants are summarized in Table 1.

**Table 1:** Baseline Patient Characteristics in International Severe Asthma Registry Study Population

Characteristic	Category	ISAR Cohort (n = 6,814)
Age, mean (SD), y	NA	55.2 (15.1)
Sex	Male	2,642 (38.8%)
	Female	4,172 (61.2%)
Smoking history	Current	307 (4.5%)
	Never	4,457 (65.4%)
	Past	2,050 (30.1%)
Ethnicity	White	5,025 (73.8%)
	Black	268 (3.9%)
	Mixed	20 (0.3%)
	NE Asian	209 (3.1%)
	SE Asian	136 (2.0%)
	Others	1,156 (16.9%)
BMI, mean (SD), kg/m^2^	NA	29.1 (6.5)
Asthma control	Not	4,210 (61.8%)
	Partial	1,678 (24.6%)
	Well	926 (13.6%)
Severe exacerbations in the past 12 mo, mean (SD)	NA	0.5 (1.2)
Asthma-related ED visits in the past 12 mo, mean (SD)	NA	0.2 (0.6)
Asthma-related hospital visits in the past 12 mo, mean (SD)	NA	0.1 (0.4)
Invasive ventilation in the past 12 mo, mean (SD)	NA	0.01 (0.1)
FEV_1_/FVC ratio, mean (SD)	NA	0.7 (0.1)
Percent predicted FEV_1_, mean (SD)	NA	73.6 (20.0)
Reversibility of FEV_1_, mean (SD)	NA	5.5 (9.1)
IgE count, mean (SD)	NA	406.7 (884.9)
Most recent Feno concentration, mean (SD), ppb	NA	41.8 (32.7)
Most recent blood eosinophil count, mean (SD), mL	NA	426.4 (326.2)
CRS and NP	None	3,329 (48.9%)
	CRS with NP	2,120 (31.1%)
	CRS without NP	1,365 (20.0%)
Eczema	Ever and current	963 (14.1%)
Anxiety	Ever and current	501 (7.4%)
Depression	Yes	514 (7.5%)
Allergic rhinitis	Yes	4,400 (64.6%)
Steroid sparing	Yes	106 (1.6 %)
Pneumonia	Yes	656 (9.6%)
Sleep apnea	Yes	933 (13.7%)
Use of LAMA	Yes	564 (8.3%)
Use of LABA/LAMA	Yes	20 (0.3%)
Use of macrolide	Yes	815 (11.9%)
Use of OCS	Yes	514 (7.5%)

Data are presented as No. (%) unless otherwise indicated. CRS = chronic rhinosinusitis; Feno = fractional exhaled nitric oxide; LABA = long-acting β_2_-agonist; LAMA = long-acting muscarinic antagonist; NA = not applicable; NE = northeast; NP = nasal polyps; OCS = oral corticosteroid; SE = southeast.

This study was approved by the institutional review board of the National University of Singapore (NUS-IRB-2021-877) and the Anonymized Data Ethics & Protocol Transparency Committee (ADEPT1924).

### Outcome Variable

The outcome, 12-month frequency of severe asthma exacerbations, was defined as an asthma attack requiring 3 or more days of OCS use, and/or the occurrence of asthma-related hospitalization or emergency room (ER) visit, aligning with the European Respiratory Society/American Thoracic Society task force definition.^22^ It was categorized into 3 groups: 0, 1, and ≥ 2 exacerbations.

### Predictor Selection

The ISAR data set included 52 patient characteristics, covering sociodemographics, lifestyles, exacerbation history, pulmonary function, biomarkers, medication use, and comorbidities (e-Appendix 2, e-Table 2). For predicting severe asthma exacerbation, we identified 28 potentially prognostic predictors (e-Appendix 3) as part of a broader project on developing risk score tools for severe asthma.^13^ These predictors were selected using expert-based scores, excluding those not universally measured or with > 50% missing data. Predictors were measured on the index date or within the immediate 12-month period before.

The BN analysis scrutinized the interaction of the full set of relevant predictors influencing future severe exacerbations. To develop a BN model for effective knowledge translation of the parallel asthma risk prediction tool,^13^ we focused on 14 key predictors selected via penalized negative binomial regression with least absolute shrinkage and selection operator (lasso). These predictors included age, sex (male, female), severe asthma exacerbation history, asthma-related ER visits, invasive ventilation, prebronchodilator measurement of FEV_1_ as a percentage of predicted value^23^ (% predicted FEV_1_), % FEV_1_ reversibility, BEC, serum IgE count, Feno, long-acting muscarinic antagonists, presence of chronic rhinosinusitis (CRS) (CRS only, CRS with nasal polyps [NPs], none), anxiety, and eczema. Of note, all ISAR patients received the combination therapy of inhaled corticosteroids and long-acting β-agonists, which was thus not considered a predictor.

### Statistical Analysis

#### Data Preprocessing

Extreme values in predictors were examined, with outliers being recoded as missing values if found (e-Appendix 4, e-Table 4). For BEC, Feno, and IgE count, any values of 0 were coded as not available (missing) and subsequently imputed (e-Appendix 4). A robust, random forest-based nonparametric method, MissForest,^24^ was used for missing value imputation in predictors with ≤ 50% missing values. Continuous predictors were discretized using information-theoretic measures, clinical thresholds, and the Hartemink^25^ criteria. To address class imbalance in the data, we applied the resampling algorithm, adaptive synthetic sampling,^26^ which generated synthetic balanced data sets, thus mitigating bias in ML methods.

#### Learning Bayesian Networks

First, we used the semi-interleaved Hiton Parents and Children (SI HITON-PC)^27^ and Max-Min Parents and Children^28^ algorithms to explore predictor interactions and arc directions among 28 relevant predictors (e-Fig 1). Second, country and residual variations in future severe exacerbations were addressed by fitting a linear mixed-effects model,^29^ incorporating predictors identified as “children” of the country variable from the local neighborhood network and the Biologic Accessibility Score^30^ as a fixed effect and country as a random effect. Third, a BN was constructed using 14 core prognostic predictors, incorporating expert knowledge with various ML algorithms (eg, Hill Climbing, Tabu Search, Peter-Clark, Grow Shrink, Restricted Maximization [general 2-phase restricted maximization]).^31^ This approach, coupled with 1,000 bootstrap iterations and a 0.75 threshold for arc selection, compensated for individual ML algorithm weaknesses, enhancing model performance. Expert input refined associations and eliminated clinically irrelevant arcs, ensuring a robust and clinically meaningful BN model.

#### Validation of the BN Model

The BN model underwent rigorous evaluation using both 10-fold cross-validation, which balances computational efficiency with reliability, and leave-1-country-out (e-Appendixes 5 and 6) cross-validation, which uses all observations to address heterogeneity. Discrimination and calibration measures were employed for model validation. Discrimination, evaluated through the area under the receiver operating characteristic curve (AUC), gauges the ability of the model to differentiate risk groups, with higher AUC values indicating superior discrimination. Calibration assesses the agreement between predicted probabilities and observed outcomes, with the calibration slope and intercept indicating model calibration. Specificity, precision, recall, and accuracy were also evaluated.

#### Conditional Probability Table, Counterfactuals, and Influence Diagram

After fitting BN to discretized data, probability distributions were estimated using ML using upstream predictor levels. Counterfactual analysis was performed by adjusting CRS levels. The final BN was transformed into an influence diagram,^21^ tackling decision problems amid uncertainty. It included chance nodes for uncertainty, decision nodes for choices, and cost/utility nodes for expenses and value linked to outcomes.

Further statistical analysis details and R packages used are listed in e-Appendixes 5 and 7.

## Results

### Patient Characteristics

Figure 1 presents the flowchart of cohort creation. The final sample included 6,814 adult patients with severe asthma who were biologic-naive during the 12-month follow-up. The mean age was 55.2 years (SD, 15.1), with 4,172 (61%) female individuals. The mean history of severe exacerbation rate in the last 12 months was 0.5 (SD, 1.2), and that of asthma-related ER visits in the last 12 months was 0.2 (SD, 0.6). In the 12-month follow-up, mean severe exacerbation rate was 0.2 (SD, 0.8). The categories of core predictors used in BN learning are presented in e-Appendix 8 and e-Table 6.

### Comprehensive Local Neighborhood Network of Relevant Predictors

**Figure 2:**
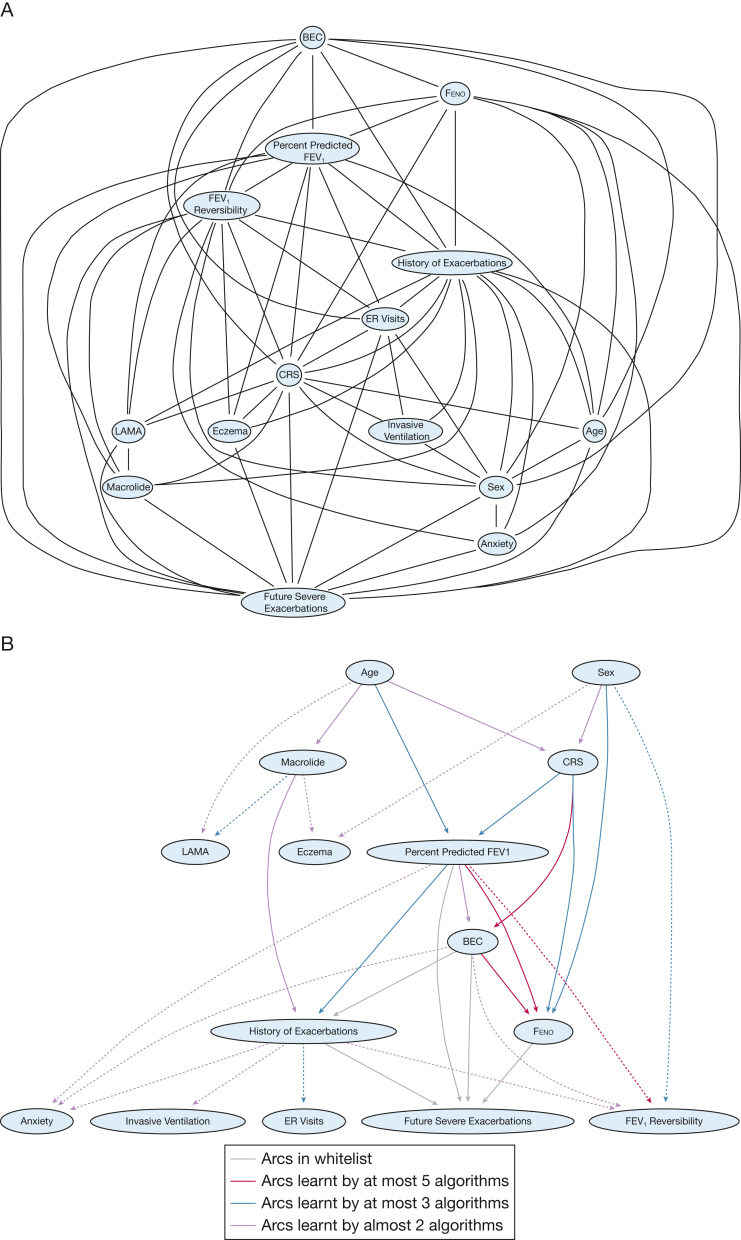

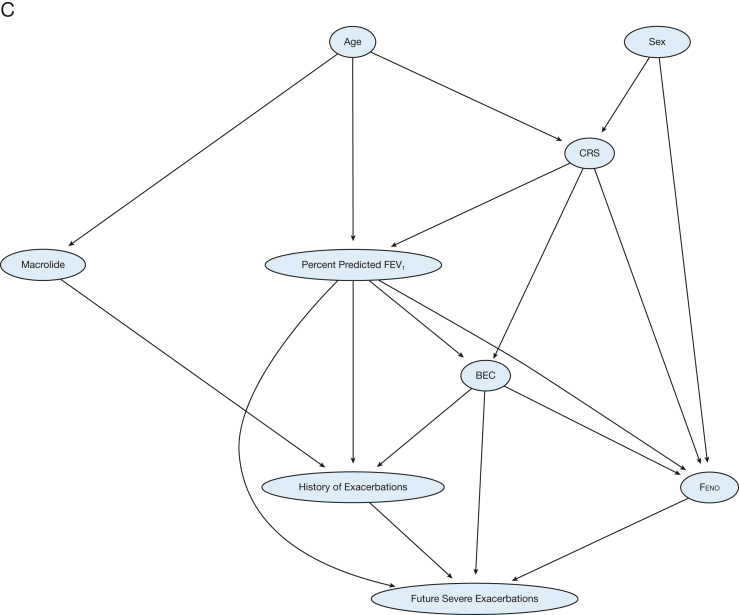
Bayesian network plots. A, Initial parent-child relationship learned; B, machine learning with expert knowledge integration; C, final model with clinically relevant arcs. BEC = blood eosinophil count; CRS = chronic rhinosinusitis; ER = emergency room; Feno = fractional exhaled nitric oxide; LAMA = long-acting muscarinic antagonist.

e-Figure 1 presents the overall local neighborhood network of 28 predictors that were expert-ranked as clinically relevant. Figure 2A displays the local neighborhood network of 14 lasso-identified essential predictors. The nodes with the most connections were the history of severe exacerbations (13 arcs), Feno (11 arcs), % predicted FEV_1_ (11 arcs), BEC (10 arcs), macrolide use (10 arcs), and CRS (6 arcs). Figure 2B shows the parameterized BN that was learned from combined ML algorithms, with CRS to BEC, % predicted FEV_1_ to Feno, and BEC to Feno as the main arcs identified by all five ML algorithms used. However, the presence of anxiety, use of invasive ventilation, asthma-related ER visits, and FEV_1_ reversibility were identified as the consequences of other predictors instead of standing in the core prediction pathway to the future severe asthma exacerbations.

### Final BN Structure Based on 14 Essential Predictors

Figure 2C presents the fine-tuned final BN in which expert knowledge was integrated. In the first pathway, history of severe exacerbations was a direct predictor of future exacerbations; meanwhile, BEC, Feno, and % predicted FEV_1_ were direct influencers of both historical and future severe exacerbations (ie, the transition from current to future frequencies of severe exacerbations). On the upper stream, the presence of CRS (with or without NP) directly influenced BEC, Feno, and % predicted FEV_1_ to affect the transition of current to future exacerbation frequencies. In the second pathway, age and sex directly influenced CRS, age directly influenced the use of macrolide, and the latter directly affected future exacerbations. Macrolide users and nonusers had notable differences in age, asthma control, prior exacerbations, medication use, and comorbidities (e-Appendix 9, e-Table 7).

### Internal-External Cross-Validation

Table 2 displays internal validation results. The predictive performance of BN model was assessed through 10-fold cross-validation and leave-1-country-out (e-Appendix 6, e-Table 5) validation, yielding AUC values of 0.65 and 0.62, respectively. Key metrics, including specificity, precision, recall, and accuracy, consistently discriminated between positive and negative cases. Using train-test data (70:30), calibration intercepts for predicting risk of ≥ 1 and ≥ 2 severe exacerbations were 0.130 and –0.018, and calibration slopes were 0.804 and 0.949 in test data, respectively, suggesting good model calibration (e-Fig 2).

**Table 2:** Internal Validation Results

Performance Metric	10-Fold Cross-Validation
AUC^a^	0.65
Specificity^b^	0.75
Precision^c^	0.50
Recall^d^	0.50
Accuracy^e^	0.63
Calibration Using 70:30 Train-Test Data	
Calibration intercept^f^	
Risk of ≥ 1 severe exacerbation	0.130
Risk of ≥ 2 severe exacerbations	–0.018
Calibration slope^f^	
Risk of ≥ 1 severe exacerbation	0.804
Risk of ≥ 2 severe exacerbations	0.949

AUC = area under the curve.

a AUC: Range between 0 and 1, indicating the probability that a classifier will rank a randomly chosen positive instance higher than a randomly chosen negative one.

b Specificity: Range between 0 and 1, measures the proportion of actual negatives that are correctly identified as such by the classifier.

c Precision: Range between 0 and 1, denotes the proportion of true positive predictions among all positive predictions made by the classifier.

d Recall: Range between 0 and 1, represents the proportion of actual positives that are correctly identified as such by the classifier.

e Accuracy: Range between 0 and 1, measures the proportion of correct predictions made by the classifier over all predictions.

f A calibration slope near 1 and an intercept close to zero signify well-calibrated models with accurate risk estimation across different individuals.

### Conditional Probability Table of BN and Counterfactual

**Figure 3:**
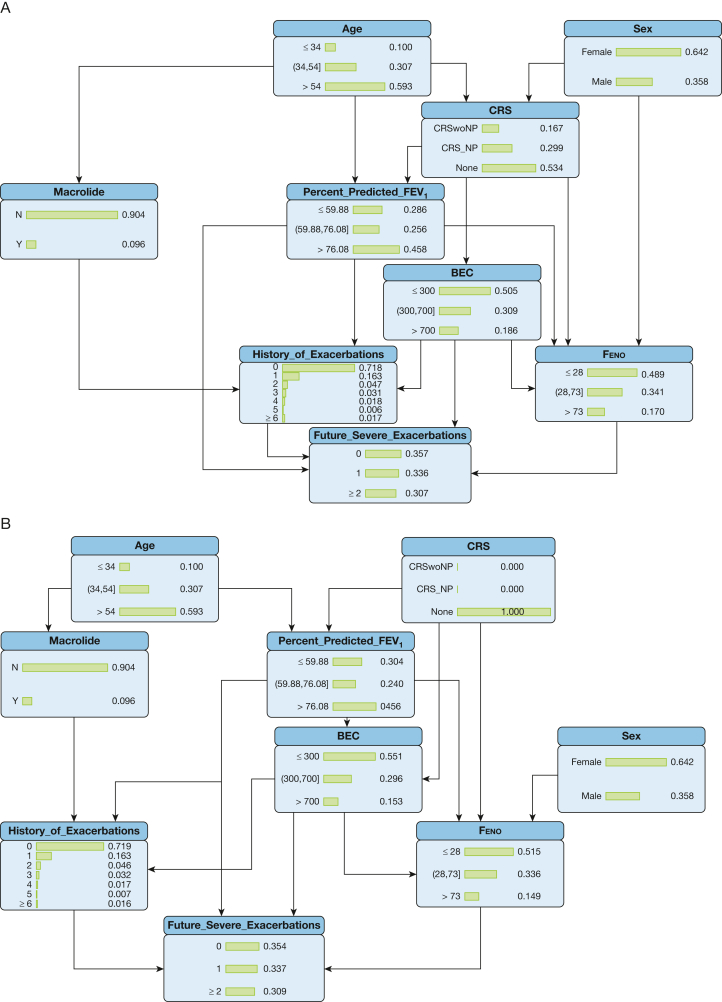

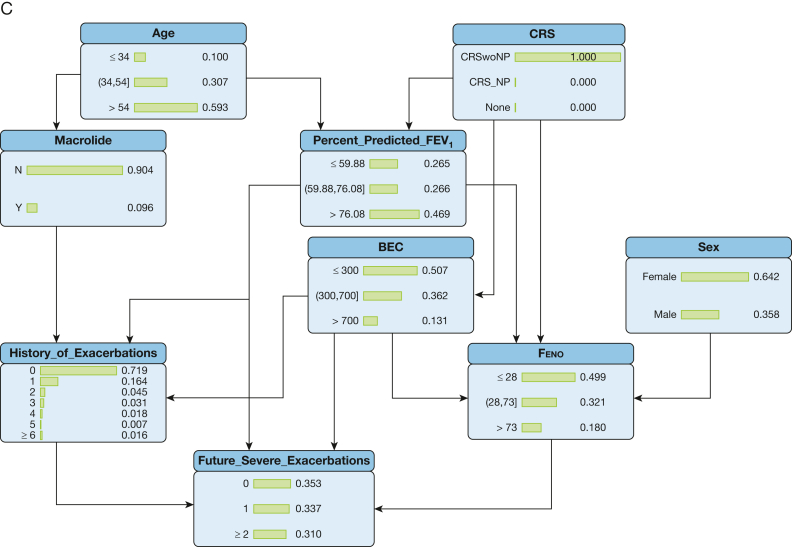
A-C, Conditional probability and counterfactual analysis. BEC = blood eosinophil count; CRS = chronic rhinosinusitis; CRS_NP = chronic rhinosinusitis with nasal polyps; CRSwoNP = chronic rhinosinusitis without nasal polyps; Feno = fractional exhaled nitric oxide.

Figure 3A illustrates the conditional probability plot for future severe exacerbations using the BN model, including different levels of upstream predictors. This conditional probability table could be used for counterfactual prediction analysis by altering node levels of predictors to affect the downstream prediction process and subsequent outcomes. For instance, in Figures 3B and 3C, by changing the CRS indicator from “None” to “CRSwoNP” (CRS without NP) the distributions of conditional probabilities of downstream predictors including % predicted FEV_1_, BEC, and Feno all subsequently changed, and thus the predicted probabilities for future severe exacerbations changed correspondingly.

### Influence Diagram

**Figure 4:**
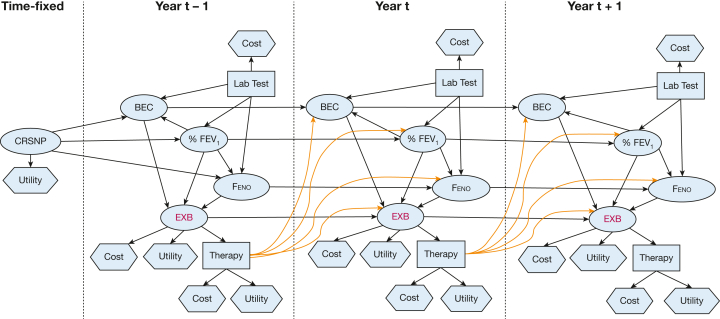
Influence diagram integrating nodes, cost, and utility. BEC = blood eosinophil count; CRSNP = chronic rhinosinusitis with nasal polyps; EXB = (severe) exacerbation; Feno = fractional exhaled nitric oxide.

Figure 4 represents the influence diagram. By removing far-distance nodes (eg, age, sex) and burden indicators (eg, medication use), this influence diagram had a particular emphasis on the influences of the presence of CRS (with or without NP) on biomarkers (BEC and Feno) and lung function (% predicted FEV_1_). In turn, % predicted FEV_1_ influenced biomarkers (BEC and Feno), and they jointly influenced the transition from current to future severe exacerbation frequencies. Treatment was integrated as a decision node (associated with utility and costs) following the events of severe exacerbation, which would influence biomarker levels and potentially lung function to affect future severe exacerbations.

## Discussion

A novel BN analysis was performed to examine the interplay of clinically relevant risk factors for asthma exacerbations in patients with severe asthma. By combining ML algorithms with expert knowledge, this study mapped the core interactive pathways directly leading to exacerbations. The BN model was developed using the largest global severe asthma registry, enhancing its generalizability.^20^ This BN analysis identified a core prediction pathway in which, under the immediate modification of CRS status, downstream predictors including lung function, BEC, and Feno directly affected the risk transition from prior to future exacerbations. A parallel prediction pathway identified macrolide use affecting prior exacerbations to predict future exacerbations. The 2 distinct pathways may correspond to different severe asthma phenotypes—early-onset symptom-predominant and late-onset inflammation-predominant, which exhibit remarkable differences in response to asthma treatment.^32^ Meanwhile, age and sex cast umbrella influences from the upper stream.

Our findings supported previous evidence on the associations between risk factors of asthma exacerbations and connected discrete evidence into coherent prediction pathways. Among patients with chronic airway diseases including asthma, those who display signs of type-2 inflammation experienced a faster decline in lung health.^33^ Among patients with eosinophilic asthma, tailored ICS and OCS doses in response to sputum eosinophils^34^ and Feno^35^ are effective in reducing severe exacerbations. The association between CRS and severe asthma has been noted,^36^ while CRS with NP is even more problematic for patients with severe asthma, being associated with further increased sputum eosinophils, Feno, and lung function impairment.^37^^,^^38^ Differential inflammatory marker concentrations across age groups have been observed in patients with CRS and those with NP,^39^ with CRS nearly twice as common in female vs male patients.^40^ Meanwhile, patients with atypical bacterial infections may experience improved symptoms and biomarkers of airway inflammation when treated with macrolide antibiotics.^41^ However, further research is required to assess the efficacy of macrolides in severe stable asthma and acute exacerbations.^42^

This study did not find IgE to be involved in the essential prediction pathway of severe exacerbations, possibly because IgE cross-linking during the acute phase might elevate eosinophil counts, contributing to the inflammatory cascade in allergic asthma without directly triggering exacerbations.^43^ Anti-IgE therapies mitigate this process by lowering free IgE, inhibiting mast cell and basophil degranulation, and decreasing inflammatory mediators, thereby reducing airway eosinophil counts, hyperresponsiveness, and remodeling, leading to fewer exacerbations.^44^^,^^45^

Similarly, lasso-selected essential predictors including anxiety, eczema, ER visits, use of invasive ventilation, and long-acting muscarinic antagonist use were identified as downstream outcomes of other predictors but were not involved in the interactive pathway that led to future exacerbations. Further, the connection of asthma control, BMI, and smoking history was explored in the structure plot taking the full set of predictors (e-Fig 1), but they were not selected by lasso as essential predictors in the asthma risk prediction model and thus were excluded from the BN analysis.

The similarity in performance metrics between 10-fold and leave-1-out cross-validations underscores the stability and robustness of the model, reinforcing its generalizability. Notably, the final BN model was associated with modest discriminative ability, whereas model calibration was good, particularly for predicting risks of recurrent exacerbations. This could be explained by the hypothesis that a single exacerbation event is a noisier indicator of the underlying disease process than multiple events. We also fitted a logistic regression and a neural network using the core predictors in the final BN, yielding AUCs of 0.58 and 0.64 for predicting ≥ 1 and ≥ 2 severe exacerbations with logistic regression and 0.58 and 0.76 with the neural network, respectively (results not included). Nonetheless, in clinical decision-making, calibration is arguably more essential than discrimination for reliably informing patients and clinicians of their actual risk.^46^ More dedicated measures beyond AUC, including net benefit, are needed to affirm the clinical utility of this BN model.^47^

This study reveals the core prediction pathways of exacerbation in patients with severe asthma. The BN also reveals heterogeneity in predicted risks, with macrolide use linked to future exacerbations through a separate pathway from the interplays between CRS, lung function, and type-2 inflammation, suggesting that patients with noneosinophilic asthma may benefit from specific risk prediction modeling. Further, by showcasing the use of a counterfactual risk prediction framework, our results encourage a “predict and prevent” approach—one that predicts the risk as well as the expected benefit from a treatment. This enables personalized interventions to alter the course of severe exacerbation episodes^11^^,^^12^ and empowers health care professionals and patients in shared decision-making. Besides, as the influence diagram integrated utility, cost nodes, and the transition of severe exacerbation frequencies in the prediction process, this study contributed to important groundwork for improving the design of cost-effectiveness analysis for advanced asthma treatments, which considers the broader impact of treatment on comorbidities including CRS, biomarker level, and lung function.

### Limitations

This study has several limitations. First, the exclusion of children and adolescents limited the generalizability of these findings to pediatric patients. In addition, among patients excluded due to insufficient follow-up period, whereas those with ≤ 30 days of follow-up in enrolled specialist clinics might not represent the target population with true severe asthma, those with 31 to 299 days of follow-up actually had similar clinical characteristics and prior histories of adverse health events as the final cohort despite lower prevalence of allergic rhinitis (54%) and CRS (40%). Second, despite the emphasis on standard measurement, the measurement of several predictors may not be consistent across ISAR countries, including medication adherence and symptom control, where the latter was measured using either GINA criteria, the Asthma Control Test, or the Asthma Control Questionnaire^48^ in different settings. Third, small-airway disease might have confounded the relationship between poor lung function and future exacerbations, whereas the ISAR data set did not capture such information.^49^^,^^50^ Fourth, essential predictors of this BN were selected through expert consensus and lasso, as per our published protocol^13^; however, adaptive or group lasso may better account for group effects among ISAR covariates. Fifth, global and temporal variations in severe exacerbation risk had been acknowledged previously,^29^ which may be influenced by diverse health care systems, unmeasured patient factors, and system-level variations. Sixth, we excluded current biologic users (12% of the ISAR data), which might introduce selection bias of “non- or low-frequency exacerbation.” However, considering the aim of this study, which aligns with the intended use of a formal risk prediction tool^13^ targeting biologic-naive patients with severe asthma, the inclusion of a biologic use indicator may complicate and bias the covariate interplays. Last, the current BN model is specific to severe asthma and lacks the ability to quantify individual prognostic values or provide clear visualizations like tables or spline curves, including the ORACLE tool.^12^

## Interpretation

This study applied a BN analysis to comprehensively assess the interactions of clinically relevant risk factors of asthma exacerbation in a large international severe asthma cohort. The findings provided significant insights for asthma risk prediction and potentially support the cost-effectiveness analysis of interventions related to type-2 inflammations and/or CRS.

## Funding/Support

This work was supported by the Singapore National Medical Research Council (Open Fund) Young Individual Research Grant (MOH-001337-00).

## Financial/Nonfinancial Disclosures

The authors have reported to *CHEST* the following: D. B. P. has advisory board membership with Amgen, AstraZeneca (AZ), Boehringer Ingelheim (BI), Chiesi, Circassia, Mylan, Mundipharma, Novartis, Regeneron Pharmaceuticals, Sanofi Genzyme, Teva Pharmaceuticals, and Thermo Fisher; consultancy agreements with Amgen, AZ, BI, Chiesi, GlaxoSmithKline (GSK), Mylan, Mundipharma, Novartis, Pfizer, Teva Pharmaceuticals, and Theravance; grants and unrestricted funding for investigator-initiated studies (conducted through Observational and Pragmatic Research Institute Pte Ltd) from AZ, BI, Chiesi, Circassia, Mylan, Mundipharma, Novartis, Pfizer, Regeneron Pharmaceuticals, Respiratory Effectiveness Group, Sanofi Genzyme, Teva Pharmaceuticals, Theravance, and the UK National Health Service; payment for lectures/speaking engagements from AZ, BI, Chiesi, Cipla, GSK, Kyorin, Mylan, Mundipharma, Novartis, Regeneron Pharmaceuticals, Sanofi Genzyme, and Teva Pharmaceuticals; payment for the development of educational materials from Mundipharma and Novartis; payment for travel/accommodation/meeting expenses from AZ, BI, Mundipharma, Mylan, Novartis, and Thermo Fisher; funding for patient enrollment or completion of research from Novartis; stock/stock options from AKL Research and Development Ltd, which produces phytopharmaceuticals; owns 74% of the social enterprise Optimum Patient Care Ltd (Australia and UK) and 74% of Observational and Pragmatic Research Institute Pte Ltd (Singapore); 5% shareholding in Timestamp, which develops adherence monitoring technology; is peer reviewer for grant committees of the Efficacy and Mechanism Evaluation Programme, and Health Technology Assessment; and was an expert witness for GSK. M. S. has received honoraria from AZ, BI, Teva, and GSK for purposes unrelated to the content of this manuscript; and has received research funding from AZ and BI directly into his research account from AZ for unrelated projects. E. W. has received honoraria from AZ, GSK, and Genentech, and has been an investigator in studies sponsored by AZ, GSK, Genentech, Sanofi, Novartis, and Teva, for which her institution has received funding. M. E. W. reports grants and/or personal fees from Novartis, Sanofi, Regeneron, Genentech, Sentien, resTORbio, Equillium, Genzyme, Cohero Health, Teva, BI, AZ, Amgen, GSK, Cytoreason, Cerecor, Sound Biologics, Incyte, and Kinaset. D. J. J. has received speaker fees and consultancy fees from AZ, GSK, Sanofi Regeneron, and BI; and has received research funding from AZ. J. B. has received research grants from AZ and personal fees from NuvoAir, outside the submitted work. L. G. H. has received grant funding, participated in advisory boards, and given lectures at meetings supported by Amgen, AZ, BI, Chiesi, Circassia, Hoffmann-La Roche, GSK, Novartis, Theravance, Evelo Biosciences, Sanofi, and Teva; has received grants from MedImmune, Novartis UK, Roche/Genentech Inc, Amgen, Genentech/Hoffman-La Roche, AZ, MedImmune, GSK, Aerocrine, and Vitalograph; has received sponsorship for attending international scientific meetings from AZ, BI, Chiesi, GSK, and Napp Pharmaceuticals; has also taken part in asthma clinical trials sponsored by AZ, BI, Hoffmann-La Roche, and GSK, for which his institution received remuneration; and is the Academic Lead for the Medical Research Council Stratified Medicine UK Consortium in Severe Asthma, which involves industrial partnerships with a number of pharmaceutical companies including Amgen, AZ, BI, GSK, Hoffmann-La Roche, and Janssen. P. E. P. has attended advisory boards for AZ, GSK, and Sanofi; has given lectures at meetings supported by AZ and GSK; has taken part in clinical trials sponsored by AZ, GSK, Novartis, and Sanofi, for which his institution received remuneration; and has a current research grant funded by GSK. D.-W. P. received sponsorships to attend or speak at international meetings, honoraria for lecturing or attending advisory boards, and research grants from the following companies: AZ, BI, GSK, Novartis, Daiichi Sankyo, Shionogi, and Orient Pharma. B. G. C. declared grants from Chiesi and GSK; personal fees for advisory board activities from Chiesi, GSK, Novartis, Sanofi, Teva, and AZ; and payment for lectures/speaking engagements from Chiesi, Novartis, GSK, Menarini, and AZ, outside the submitted work. L. P.-d.-L. reports grants, personal fees, and nonfinancial support from AZ; personal fees and nonfinancial support from GSK; grants, personal fees, and nonfinancial support from TEVA; personal fees and nonfinancial support from Chiesi; grants, personal fees, and nonfinancial support from Sanofi; personal fees from MSD; personal fees from Techdow Pharma; grants, personal fees, and nonfinancial support from FAES; personal fees from Leo-Pharma; grants and personal fees from GEBRO; and personal fees from GILEAD, outside the submitted work. R. A.-L. has given lectures at meetings supported by AZ, BI, Novartis, GSK, and Sanofi; and participated in advisory board fees from GSK, AZ, Novartis, and Abbot. D. L. L. reports personal fees from ALK-Abelló, AZ (national and global), Bayer, Chiesi, Grunenthal, Grin, GSK (national and global), Viatris, Menarini, MSD, Novartis, Pfizer, Sanofi, Siegfried, UCB, and Carnot; and grants from AbbVie, Bayer, Lilly, Sanofi, AZ, Pfizer, Novartis, Circassia, UCB, and GSK, outside the submitted work. M. S. A.-A. has received advisory board and speaker fees from AZ, Sanofi, Novartis, and GSK; and received a grant from the Kuwait Foundation for the Advancement of Sciences (KFAS). C. K. R. has received consulting/lecture fees from MSD, AZ, GSK, Novartis, Takeda, Mundipharma, BI, Teva, Sanofi, and Bayer. T. I. received lecture fees from Kyorin, GSK, Novartis, BI, and AZ. E. H. declares personal fees from Sanofi, Regeneron, GSK, Novartis, AZ, Stallergenes, and Circassia. G. W. C. has received research grants, as well as lecture or advisory board fees, from A. Menarini, ALK-Abelló, Allergy Therapeutics, Anallergo, AZ, MedImmune, BI, Chiesi Farmaceutici, Circassia, Danone, Faes, Genentech, Guidotti Malesci, GSK, Hal Allergy, Merck, MSD, Mundipharma, Novartis, Orion, Sanofi Aventis, Sanofi, Genzyme/Regeneron, Stallergenes, UCB Pharma, Uriach Pharma, Teva, Thermo Fisher, and Valeas. R. W. C. has received honoraria for lectures from Aerogen, AZ, BI, GSK, Novartis, and Teva; is a member of advisory boards for GSK and Novartis; has received grant support from GSK and Aerogen; and has patents in the use of acoustics in the diagnosis of lung disease, assessment of adherence, and prediction of exacerbations. N. G. P. has been a speaker and/or advisory board member for Abbott, AbbVie, ALK, Asit Biotech, AZ, Biomay, BI, GSK, HAL, Faes Farma, Medscape, Menarini, MSD, Novartis, Nutricia, OM Pharma, Regeneron, Sanofi, Takeda, and Viatris. A. I. P. has received fees and honoraria from Menarini, GSK, Novartis, Elpen, BI, AZ, and Chiesi. C. M. P. has attended advisory boards for AZ, Novartis, TEVA, and Sanofi-Genzyme; has given lectures at meetings supported by AZ, Novartis, TEVA, Sanofi-Genzyme, and GSK; has taken part in clinical trials sponsored by AZ, Novartis, MSD, Sanofi-Genzyme, GSK, and Novartis; and has received educational and research grants from AZ, Novartis, TEVA, GSK, ALK, and Sanofi-Genzyme. C. A. T.-D. has received fees as advisory board participant and/or speaker from AZ, BI, GSK, Novartis, and Sanofi-Aventis; has taken part in clinical trials from AZ, Novartis, and Sanofi-Aventis; and has received unrestricted grants for investigator-initiated studies at Fundación Neumológica Colombiana from AZ, BI, GSK, Grifols, and Novartis. T. A. P. declares relevant research support from Novartis and Chiesi Pharma. M. H. declares grants and other advisory board fees (made to his institutional employer) from AZ, GSK, Novartis, Sanofi, Teva, and Seqirus, for unrelated projects. M. J. P. declares personal fees and nonfinancial support from AZ and GSK. P. G. G. has received speaker fees and grants to his institution from AZ, GSK, and Novartis. J. M. reports speaker fees, grants, or advisory boards for AZ, Sanofi, GSK, Novartis, Inmunotek, Menarini, and Noucor. C. B. reports advisory board participation of Sanofi, AZ, Takeda, and Valeo Pharma; and honoraria for presentations for GSK, AZ, Amgen, Grifols, Sanofi, Regeneron, and Valeo Pharma. S. C. declares receiving conference fees from Novartis SA de CV, GSK Mexico, AZ Mexico, and Sanofi Mexico. None declared (C. P. Y., A. C., L. H. M. L., Y. R. J., R. B., C. J., M. K. S., B. M., G. C. C., E. A. C., W. C.).

## Supplementary Data

e-Online Data
