# Supplementary material for: Prediction Pathway for Severe Asthma Exacerbations: A Bayesian Network Analysis
Source: Chest. 2025 May 19;168(2):301–16. doi: 10.1016/j.chest.2025.04.046 (PMC12405933; doi:10.1016/j.chest.2025.04.046)
Supplement: e-Online Data [file mmc1.docx]

**Prediction pathway for severe asthma exacerbations: a Bayesian Network analysis**

**Supplementary Materials**

**Table of Contents**

[**1. e-Appendix 1: Diagnostic Criteria for Asthma and Definition of Severe Asthma across Countries** 1](#_Toc201830506)

[**2. e-Appendix 2: Full List of Predictors as per Different Groups** 8](#_Toc201830507)

[**3. e-Appendix 3: Full Set of Predictors** 11](#_Toc201830508)

[**4. e-Appendix 4: Missing Data Summary Tables** 11](#_Toc201830509)

[**5. e-Appendix 5: Detailed Statistical Analysis Plan** 12](#_Toc201830510)

[**6. e-Appendix 6: Leave-One-Country-Out Cross Validation and Additional Figures** 14](#_Toc201830511)

[**7. e-Appendix 7: Packages Used for BN Model Development in R** 15](#_Toc201830512)

[**8. e-Appendix 8: Predictors with Categories Discretized using Hartemink Criteria and Clinical Cutoffs** 15](#_Toc201830513)

[**9. e-Appendix 9: Characteristics of Macrolide Users vs Non-Users Sample** 17](#_Toc201830514)

[**10. References** 24](#_Toc201830515)

### **1. e-Appendix 1: Diagnostic Criteria for Asthma and Definition of Severe Asthma across Countries**

**e-Table 1: A summary of how each registry diagnoses asthma and categorizes severe asthma.**

| Country | Severe Asthma Definition* | Diagnosis Confirmation | Age of asthma onset |
| --- | --- | --- | --- |
| Argentina  Bulgaria  Canada  Greece  India  Ireland  Japan  Kuwait  Saudi Arabia  South Korea  Taiwan  United Arab Emirates | ERS/ATS Task Force guidelines on assessment and treatment of severe asthma, based on GINA guidelines for asthma control; a stepwise approach towards pharmacotherapy.  1)  GINA Step 5 if:  At least one of the following:  a) Anti-immunoglobulin E (omalizumab)  b) Anti-interleukin-5 (mepolizumab, reslizumab, benralizumab)  c) Anti-interleukin-4 (dupilumab)  d) Bronchial thermoplasty,  e) Maintenance oral corticosteroids.  OR  2)  a) GINA Step 4 if:  Medium to High dose ICS plus second controller (LABA AND/OR LAMA)  WITH/WITHOUT Extra controller (e.g. leukotriene receptor antagonist (LTRA) AND/OR theophylline)  AND  b) Uncontrolled asthma if at least one of the following is fulfilled:   - Poor symptom control:   - Asthma Control Questionnaire (ACQ) >1.5, OR   - Asthma Control Test (ACT) <20, OR   - Having at least 3 of the following during the past 4 weeks (NAEPP/GINA guidelines):     - - Daytime symptoms >twice/week,       - Night waking due to asthma,       - Reliever needed >twice/week,       - Activity limitation due to asthma - Airflow limitation: FEV1< 80% predicted (in the face of reduced FEV1/FVC following a withhold of short and long-acting bronchodilators, i.e. Pre-bronchodilator) - Serious exacerbations: at least one hospitalization, ICU stay or mechanical ventilation in the previous year - Frequent severe asthma exacerbations: two or more short courses of systemic corticosteroids (>3 days course each in the previous year) | 1) Clinical diagnosis of severe asthma  OR  2) Clinical opinion by physicians (according to severe asthma definition) | 1) Age at which asthma is first diagnosed  OR  2) Age at which asthma symptoms were first observed  (Whichever occurred first) |
| Australia | 1) Uncontrolled on GINA Step 4 Treatment  -ERS/ATS guideline for uncontrolled asthma:  a) Poor symptom control: ACQ consistently >1.5, ACT< 20 (or 'not well controlled' by NAEPP/GINA guidelines) AND/OR  b) Frequent severe exacerbations: 2 or more bursts of systemic CSs (>3 days each) in the previous year AND/OR  c) Serious exacerbations: at least one hospitalization, ICU stay or mechanical ventilation in the previous year AND/OR  d) Persistent airflow limitation: FEV1< 80% predicted (in the face of reduced FEV1/FVC following a withhold of short and long-acting bronchodilators (i.e. PRE-bronchodilator) | Airflow Obstruction:  1) Bronchodilator Response (BDR) > 200 mL and/or > 12%  2) Airway hyperresponsiveness (AHR) in response to any standard challenge agent  3) Peak flow variability >12%  4) FEV_1_ Variability >12% | 1) Age when asthma symptoms began  2) Age at which asthma was first diagnosed  3) Age of first use of asthma treatment |
| Colombia | 1) GINA Step 4 uncontrolled or GINA Step 5  -ERS/ATS guideline for uncontrolled asthma:  a) Poor symptom control: ACQ consistently >1.5, ACT < 20 (or 'not well controlled' by NAEPP/GINA guidelines) AND/OR  b) Frequent severe exacerbations: 2 or more bursts of systemic CSs (>3 days each) in the previous year AND/OR  c) Serious exacerbations: at least one hospitalization, ICU stay or mechanical ventilation in the previous year AND/OR  d) Persistent airflow limitation: FEV1< 80% predicted (in presence of reduced FEV1/FVC following a withhold of short and long-acting bronchodilators | 1) A clinical diagnosis of asthma plus functional confirmation:  a) Bronchodilator Response (BDR) > 200 mL and > 12%, or  b) Airway hyperresponsiveness (AHR) in response to any standard challenge agent. or  c) FEV1 Variability >12% or PEF variability > 20%  2) If functional confirmation was not obtained, a clinical diagnosis of asthma done by a pulmonologist was accepted. | 1) Age of onset of asthmatic symptoms  2) Before age of 12 or after age of 12 |
| Denmark  Mexico | 1) GINA Step 5 Treatment  2) Uncontrolled on GINA Step 4 Treatment  -ERS/ATS guideline for uncontrolled asthma:  a) Poor symptom control: ACQ consistently >1.5, ACT< 20 (or 'not well controlled' by NAEPP/GINA guidelines) AND/OR  b) Frequent severe exacerbations: 2 or more bursts of systemic CSs (>3 days each) in the previous year AND/OR  c) Serious exacerbations: at least one hospitalization, ICU stay or mechanical ventilation in the previous year AND/OR  d) Persistent airflow limitation: FEV1< 80% predicted (in the face of reduced FEV1/FVC following a withhold of short and long-acting bronchodilators (i.e. PRE-bronchodilator) | NIL | Age at patient's first asthma symptoms |
| Italy | 1) ERS/ATS Severe Asthma definition:  Asthma that requires treatment with guidelines suggested medications for GINA steps 4–5 asthma for the previous year or systemic CS for ≥50% of the previous year to prevent it from becoming “uncontrolled” or remaining “uncontrolled” despite this therapy | 1) Method of diagnosis:  (a) Methacholine  (b) Bronchodilation  (c) Functional Respiratory Variability | 1) Age of asthma onset  2) Age at diagnosis |
| Spain | 1) GEMA Step 5 or Step 6 Treatment  2) Uncontrolled on GEMA Step 4 Treatment:  a) Symptoms of severe asthma  b) Frequent exacerbations that require the use of systemic corticosteroids | NIL | Year of asthma diagnosis |
| UK | 1) ERS/ATS Severe Asthma definition:  Asthma that requires treatment with guidelines suggested medications for GINA steps 4–5 asthma for the previous year or systemic CS for ≥50% of the previous year to prevent it from becoming “uncontrolled” or remaining “uncontrolled” despite this therapy | NIL | Date first seen by the chest physician |

*All patients included in the current study met ISAR’s standardized definition of severe asthma

ACQ: Asthma Control Questionnaire; ACT: Asthma Control Test; A&E: Accident & Emergency; AHR: airway hyper-responsiveness; ATS: American Thoracic Society; BG: Bulgaria; BTS: British Thoracic Society; CN: Canada; DK: Denmark; ERS: European Respiratory Society; ES: Spain; FEV1: forced expiratory volume in 1 second; FVC: forced vital capacity; GINA: Global Initiative for Asthma; GR: Greece; ICS: inhaled corticosteroid; ICU: Intensive Care Unit; ISAR: International Severe Asthma Registry; IT: Italy; JP: Japan; KW: Kuwait; LABA: long-acting β2-agonist; LAMA: long-acting muscarinic antagonist; LTRA: leukotriene receptor antagonist; NAEPP: National Asthma, Education, and Prevention Programme; NICE: National Institute for Clinical Excellence; OCS: oral corticosteroid; pMDI: pressurised metered dose inhaler; SK: South Korea.

* GINA confirmed asthma diagnosis with confirmed variable airflow obstruction current or historical within 10 years:

- Bronchodilator response > 200 mL or >12% (post-bronchodilator FEV1 following administration of 400 µg salbutamol, pMDI with spacer after 10 mins AND/OR
- AHR in response to any standard challenge agent (e.g. methacholine, histamine, hypertonic saline, mannitol, adenosine monophosphate, exercise) AND/OR
- Peak flow variability > 12% over at least 1 week AND/OR FEV1 variability >12% within 2 months

Reference GEMA 4.0: Guía Española para el manejo del asma (Spanish Guideline for Asthma Management). Plaza Moral V; Comité Ejecutivo de GEMA. [GEMA(4.0). Guidelines for Asthma Management. Arch Bronconeumol. 2015 Jan;51 Suppl 1:2-54. doi: 10.1016/S0300-2896(15)32812-X. Diagnostic criteria: Asthma symptoms plus: positive bronchodilator response, or PEF variability, or exhaled nitric oxide > 50 ppb, or bronchial hyperresponsiveness, or complete reversion of bronchial obstruction after an oral corticosteroid test.

### **2. e-Appendix 2: Full List of Predictors as per Different Groups**

**e-Table 2. Full list of ISAR variables that will be considered as potential predictors in the online survey.**

| Group | Variable | Definition |
| --- | --- | --- |
| Patient characteristics | Age | Age of the patient. |
|  | Sex | Gender of the patient. |
|  | Ethnicity | Which ethnic group does the patient belong to? |
|  | Smoking Status | Whether the patient has ever smoked. |
|  | BMI | Body Mass Index (BMI) = Weight (kg)/(Height (m))^2 of the patient. |
|  | Asthma Control | Defined and categorized by GINA 2022 assessment of asthma symptom control, Asthma Control Test, or Asthma control questionnaire |
|  | Duration of Asthma | Time duration of the affliction (Age - Asthma onset age). |
| Timing | Season | The country-wise prevalent season during the index month. |
| Pulmonary function tests | Pre-bronchodilator FEV1 | Pre-bronchodilator measure of forced expiratory volume at 1second (FEV1) |
|  | Pre-bronchodilator FVC | Pre-bronchodilator measure of forced vital capacity (FVC) |
|  | Pre-bronchodilator FEV1/FVC Ratio | Pre-bronchodilator ratio of FEV1 and FVC. |
|  | Post-bronchodilator FEV1 | Post-bronchodilator FEV1 measure. |
|  | Post-bronchodilator FVC | Post-bronchodilator FVC measure. |
|  | Post-bronchodilator FEV1/FVC Ratio | Post-bronchodilator ratio of FEV1 and FVC. |
|  | Fractional Exhaled  Nitric Oxide (FeNO) | Fraction Exhaled Nitric Oxide Test result. |
| Biomarkers | Baseline Eosinophil  Counts (BEC) | Highest blood eosinophil count in the last year. |
|  | IgE count | Total serum IgE count within the last year. |
|  | Composite Eosinophilic Gradient | Calculated based on biologics use, eosinophil counts & FeNO results^51^. |
| Positive allergen tests | Serum specific IgE test to allergens | Whether the patient has tested positive to any of a number of serum Allergen Test of Grass Mix, Weed Mix, Mould Mix, Dust Mite, Cat, Dog , Trees, Aspergillus, Food Mix, Animal Mix, Asperillus, Other   - Categorized as a positive reaction if >0.7kU/L |
|  | Skin prick test to allergens | Whether the patient has tested positive to Skin prick test to house dust mite, animal dander (cat, dog), pollen (tree, grass) and moulds (Aspergillus).  Categorized as a positive reaction if >4 mm is wheal Diameter |
| Health services use | Exacerbations | Total number of exacerbations occurring in the last year |
|  | Total ER visits | Total asthma-related Emergency Room (ER) visits in the past year |
|  | Total Hospital visits | Total asthma-related cases of hospitalization within the past year |
|  | Invasive Ventilation | Total number of invasive ventilations within the past year |
|  | Medication adherence | Whether the patient has evidence of poor medical adherence |
| OCS use | Long-term OCS | Whether the patient takes long-term/maintenance oral corticosteroids (OCS) |
| Other medication use (all patients were required to be taking medium or high dose ICS/LABA) | LAMA | Whether the patient takes long-acting muscarinic antagonists (LAMA) |
|  | LABA/LAMA | Whether the patient takes a combination therapy of long-acting beta-agonist (LABA) and LAMA |
|  | Macrolide | Whether the patient takes long-term macrolide |
|  | Steroid sparing | Whether the patient takes steroid-sparing drugs such as biologic therapies or immunomodulators. |
| Comorbidities | Allergic rhinitis | Did this patient have a positive diagnosis of allergic rhinitis as of or prior to the baseline visit? |
|  | Chronic rhinosinusitis | Did this patient have a positive diagnosis of Chronic rhinosinusitis of or prior to the baseline visit? |
|  | Nasal polyps | Did this patient have a positive diagnosis of Nasal polyps of or prior to baseline visit? |
|  | Eczema | Did this patient have a positive diagnosis of Eczema of or prior to baseline visit? |
|  | GERD | Did this patient have a positive diagnosis of GERD of or prior to baseline visit? |
|  | Bronchiectasis | Did this patient have a positive diagnosis of Bronchiectasis of or prior to baseline visit? |
|  | COPD | Did this patient have a positive diagnosis of COPD of or prior to baseline visit? |
|  | Anxiety | Did this patient have a positive diagnosis of Anxiety of or prior to baseline visit? |
|  | Depression | Did this patient have a positive diagnosis of Depression of or prior to baseline visit? |
|  | Dysfunctional breathing | Did this patient have a positive diagnosis of Dysfunctional breathing of or prior to baseline visit? |
|  | Hypertension | Did this patient have a positive diagnosis of Hypertension of or prior to baseline visit? |
|  | Pneumonia | Did this patient have a positive diagnosis of Pneumonia of or prior to baseline visit? |
|  | Cataract | Did this patient have a positive diagnosis of Cataract of or prior to baseline visit? |
|  | Glaucoma | Did this patient have a positive diagnosis of Glaucoma of or prior to baseline visit? |
|  | Renal Failure | Did this patient have a positive diagnosis of Renal Failure of or prior to baseline visit? |
|  | Heart failure | Did this patient have a positive diagnosis of Heart failure of or prior to baseline visit? |
|  | IHD | Did this patient have a positive diagnosis of IHD of or prior to baseline visit? |
|  | Embolism | Did this patient have a positive diagnosis of Embolism of or prior to baseline visit? |
|  | Peptic Ulcer | Did this patient have a positive diagnosis of Peptic Ulcer of or prior to baseline visit? |
|  | Type II Diabetes | Did this patient have a positive diagnosis of Type II Diabetes of or prior to baseline visit? |
|  | Sleep Apnea | Did this patient have a positive diagnosis Sleep Apnea of or prior to baseline visit? |
|  | Stroke | Did this patient have a positive diagnosis of Stroke of or prior to baseline visit? |
|  | Obesity | Did this patient have a positive diagnosis of Obesity of or prior to baseline visit? |

### **3. e-Appendix 3: Full Set of Predictors**

These shortlisted predictors were categorized into patient characteristics including age, gender (male, female), ethnicity (Caucasian, African, Mixed, North East Asian, Others, South East Asian), body mass index (BMI), smoking history (current, never, past), asthma control (controlled, partially controlled, uncontrolled), history of severe exacerbations, pulmonary function tests including pre-bronchodilator measurement of forced expiratory volume in 1 second as percentage of predicted value^1^ (% predicted FEV1), pre-bronchodilator measurement of the ratio of FEV1 to the forced vital capacity of the lungs (FEV1/FVC), percent reversibility of post-over pre-bronchodilator FEV1 values; biomarkers including blood eosinophil count (BEC), serum Immunoglobulin E (IgE) count, fractional exhaled nitric oxide (FeNO), health services used (HSU) including asthma-related hospitalizations and emergency room (ER) visits, use of invasive ventilation, long term use of oral corticosteroids (OCS), other medication use including use of steroid-sparing medications, long-acting muscarinic antagonists (LAMA), combination therapy with long-acting beta-agonists and LAMA (LABA-LAMA), macrolide, comorbidities including presence of chronic rhinosinusitis (CRS only, CRS with nasal polyps (NP), none), allergic rhinitis, anxiety, depression, eczema, pneumonia, obstructive sleep apnea.

### **4. e-Appendix 4: Missing Data Summary Tables**

This section lists the missing observations available as predictors within the study sample (N = 6,814). Notably, this study aligns with our previously published individualized risk prediction protocol^5^, in which we used specific criteria for missing data imputation, applying it to predictors with ≤50% missing information. The data were prepared following the protocol, and the study sample was selected based on the inclusion and exclusion criteria outlined in the patient flowchart. This explains why some predictors in the study sample have more than 50% missingness, even though this was not the case in the original dataset.

**e-Table 4: Missing data summary for predictors included in the study population**

| **Predictors** | **Missing N (%)** |
| --- | --- |
| Age | 0 (0%) |
| Smoking History | 1533 (23%) |
| Ethnicity | 0 (0%) |
| BMI | 1501 (22%) |
| Asthma Control | 2065 (30%) |
| History of Severe Asthma Exacerbations | 0 (0%) |
| No. of emergency room visits | 0 (0%) |
| No. of hospital visits | 0 (0%) |
| Invasive ventilation | 0 (0%) |
| Pre-bronchodilator EV1 | 1657 (24%) |
| Pre-bronchodilator FEV1/FVC | 1679 (25%) |
| IgE count | 3030 (44%) |
| FeNO | 3127 (46%) |
| Blood eosinophil count | 2042 (30%) |
| Chronic rhinosinusitis | 4028 (59%) |
| Nasal polyps | 4163 (61%) |
| Eczema | 4140 (61%) |
| Anxiety | 4165 (61%) |
| Depression | 4169 (61%) |
| Allergic rhinitis | 4364 (64%) |
| Steroid sparing | 0 (0%) |
| Pneumonia | 4475 (0%) |
| Sleep apnea | 3911 (57%) |
| LAMA | 0 (0%) |
| LABA/LAMA | 0 (0%) |
| Macrolide | 0 (0%) |
| Use of long-term OCS | 927 (14%) |

### **5. e-Appendix 5: Detailed Statistical Analysis Plan**

**Missing data imputation**

For predictors with ≤50% missing values, we employed MissForest, a robust, non-parametric, random-forest-based algorithm, to impute missing data^2^. The descriptive table for missing data as per each variable is reported in **e-Table 5**.

**Discretization of continuous predictors**

Bayesian networks (BNs) encounter challenges when dealing with continuous variables, often necessitating discretisation for easier management. Discretization permits the utilisation of existing methods and broadens the applicability for inference and learning of BNs, albeit resulting in an approximation. We discretised continuous predictors using information-theoretic measures, clinically relevant cutoff thresholds, and the Hartemink criteria^3^.

**Resampling to address data imbalance**

Machine learning (ML) classifiers often favor the majority class when dealing with highly imbalanced datasets. Instead of allocating additional resources to gather more data, resampling proved to be a good solution, which extracts as much valuable information as possible from the existing dataset. We applied the ADASYN algorithm to generate a synthetic balanced dataset^4^ to mitigate the imbalance issue in the ISAR data.

**Learning Bayesian networks**

First, to understand the comprehensive environment of predictor interactions and the direction of arcs, we employed a constraint-based algorithm, semi-interleaved HITON-PC (SI HITON-PC), on the full list of 28 relevant predictors to identify local neighbours of each predictor and indicate which other predictors might influence (“parents”) or be influenced (“children”) by this current predictor along with the Max-Min Parents and Children (MMPC) algorithm. A related structure plot is given in **e**-**Figure 1**.

Second, we adjusted for country variations and unexplained or residual variations in future severe exacerbations by fitting a linear mixed-effects model. The fixed effect included all predictors identified as the “children” of the country variable from the above-learned local neighborhood network, and the Biologic Accessibility Score and random effect included the country.

Third, we used 14 core prognostic predictors to learn BN. Model parameterization involved combining expert knowledge (utilizing whitelist/blacklist for some arcs) with ML algorithms: scoring (Hill Climbing, Tabu Search), constraint-based (Peter-Clark, Grow Shrink), and hybrid (RSMAX2) with 1000 bootstrap iterations and a 0.75 threshold for BN arc selection. This approach compensated for individual ML algorithm weaknesses, significantly enhancing model performance. Expert input refined associations and eliminated clinically irrelevant arcs, ensuring a robust and clinically meaningful BN model.

**Validation of the BN model**

The BN model was evaluated using 10-fold cross-validation and leave-one-country-out cross-validation. In 10-fold cross-validation, data was divided into 10 subsets, with each subset used for model validation while training on the remaining 9 subsets. Leave-one-country-out cross-validation excluded one country at a time from the training set to assess performance across diverse country-specific datasets.

Discrimination and calibration measures were used for model validation. Discrimination is the ability of a model to separate patients into different risk groups. An area under the receiver operating characteristic curve (AUC) value closer to 1 indicates excellent discrimination, meaning the model can perfectly distinguish between positive and negative cases. An AUC closer to 0.5 suggests poor discrimination, where the model performs no better than random guessing.

Calibration assesses the agreement between predicted probabilities and observed outcomes. The calibration slope measures how well predicted probabilities match actual outcomes; closer to 1 indicates good calibration. The calibration intercept, also called calibration-in-the-large, assesses whether the model's average predicted risk matches the overall observed event rate; closer to 0 indicates good calibration. Calibration plots with a 70:30 train-test split were employed to ensure model reliability across various datasets.

Other crucial statistics included specificity, precision, recall, and accuracy. The models were expected to have high accuracy, precision, recall, and AUC, along with well-calibration.

**Some assumptions needed to be made about the validity of the BN model**

To ensure the validity of the model, we make the following assumptions:

1. Age, gender, ethnicity, and smoking history cannot depend on any other predictors.
2. The outcome, future severe exacerbation, cannot have a causal impact on any other predictors.
3. The future severe exacerbation is the output we are interested in and represents the end node of the BN.

### **6. e-Appendix 6: Leave-One-Country-Out Cross Validation and Additional Figures**

**e-Table 5: Internal validation results (leave-one-country-out cross-validation)**

| Performance Metrices | leave-one-country-out validation |
| --- | --- |
| AUC^1^ | 0.62 |
| Specificity^2^ | 0.74 |
| Precision^3^ | 0.49 |
| Recall^4^ | 0.49 |
| Accuracy^5^ | 0.62 |

AUC (Area Under the Curve): Range between 0 and 1, indicating the probability that a classifier will rank a randomly chosen positive instance higher than a randomly chosen negative one.

Specificity: Range between 0 and 1, measures the proportion of actual negatives that are correctly identified as such by the classifier.

Precision: Range between 0 and 1, denotes the proportion of true positive predictions among all positive predictions made by the classifier.

Recall: Range between 0 and 1, represents the proportion of actual positives that are correctly identified as such by the classifier.

Accuracy: Range between 0 and 1, measures the proportion of correct predictions made by the classifier over all predictions.

### **7. e-Appendix 7: Packages Used for BN Model Development in R**

1. **bnstruct**: Provides functions for learning Bayesian networks from data.
2. **bnlearn**: Facilitates learning the structure and parameters of Bayesian networks from data.
3. **caret**: Offers a comprehensive set of tools for training and evaluating machine learning models.
4. **gRain**: Facilitates working with graphical models, including Bayesian networks.
5. **nlme**: Implements nonlinear mixed-effects models for hierarchical data structures.
6. **Rgraphviz**: Enables visualization of graph objects and networks.
7. **UBL**: Provides utilities for balancing and synthesizing imbalanced datasets.
8. **rms**: The val.prob() function is used to compute predicted probabilities from a model fit using the validate() function.

### **8. e-Appendix 8: Predictors with Categories Discretized using Hartemink Criteria and Clinical Cutoffs**

**e-Table 6: Categories of core risk predictors used in the construction of BN.**

| **Predictors** | **Categories** |
| --- | --- |
| BEC | <=300, (300, 700], >700 |
| FeNO | <=28, (28, 73], >73 |
| IgE Count | <=36, (36, 240], >240 |
| Percent Predicted FEV1 | <=59.88, (59.88, 76.08], >76.08 |
| The ratio of FEV1 to FVC | <=0.57, (0.570.70], >0.70 |
| FEV1 Reversibility | <=0.92, (0.92, 10.62], >10.62 |
| Baseline Exacerbation | 0, 1, 2, 3, 4, 5, >=6 |
| Hospital Visit | 0, 1, >=2 |
| ER Visit | 0, 1, 2, >=3 |
| BMI | underweight, normal, overweight, obese |
| Invasive Ventilation | 0, >=1 |
| Age | <=34, (34, 54], >54 |
| Future Exacerbation | 0, 1, >=2 |
| Sex | Female, Male |
| Sleep apnea | N, Y |
| Pneumonia | N, Y |
| Eczema | N, Y |
| Ethnicity | Caucasian, African, Mixed, NE Asian, Others, SE Asian |
| Depression | N, Y |
| Macrolide | N, Y |
| Anxiety | N, Y |
| LAMA | N, Y |
| LABA/LAMA | N, Y |
| Steroid sparing | N, Y |
| Allergic rhinitis | N, Y |
| Use of long-term OCS | N, Y |
| Smoking History | Current, Never, Past |
| Asthma Control | Not, Partial, Well |

### **9. e-Appendix 9: Characteristics of Macrolide Users vs Non-Users Sample**

Key points to be noted based on the macrolide users sample:

- Macrolide users tend to be older and have more comorbid conditions (CRS, eczema, depression, allergic rhinitis, pneumonia, sleep apnea).
- They show better asthma control and lung function with fewer previous exacerbations, higher future exacerbations, ER visits, and hospitalizations.
- They have lower BEC, lower IgE, and lower FEV1 reversibility, suggesting a different inflammatory phenotype.
- They are more likely to be on steroid-sparing therapy, LAMA, LABA/LAMA, and long-term OCS, indicating they might have been prescribed macrolides as an add-on therapy for difficult-to-treat asthma.

e-Table 6: Patient characteristics of macrolide users vs non-users based on study sample (N = 6814)

| **Variables** |  | **Non-Macrolide Users**  **(N=5999)** | **Macrolide Users**  **(N=815)** | **P value** |
| --- | --- | --- | --- | --- |
| Age (years), mean(SD) |  | 54.65 (15.05) | 59.49 (15.05) | 0 |
| History of Smoking, n(%) | Current | 268 (4.47%) | 39 (4.79%) | 0.63 |
|  | Past | 1795 (29.92%) | 255 (31.29%) |  |
|  | Never | 3936 (65.61%) | 521 (63.93%) |  |
| Ethnicity, n(%) | African | 224 (3.73%) | 44 (5.40%) | 5e-04 |
|  | Caucasian | 4425 (73.76%) | 600 (73.62%) |  |
|  | NE Asian | 201 (3.35%) | 8 (0.98%) |  |
|  | Others | 1008 (16.80%) | 148 (18.16%) |  |
|  | SE Asian | 121 (2.02%) | 15 (1.84%) |  |
|  | Mixed | 20 (0.33%) | 0 (0%) |  |
| BMI (kg/m^2), mean(SD) |  | 29.06 (6.45) | 29.44 (6.61) | 0.12 |
| Asthma Control, n(%) | Not | 3753 (62.56%) | 457 (56.07%) | 0.001 |
|  | Partial | 1441 (24.02%) | 237 (29.08%) |  |
|  | Well | 805 (13.42%) | 121 (14.85%) |  |
| History of severe asthma exacerbation, mean(SD) |  | 0.55 (1.29) | 0.44 (0.72) | 0 |
| Future severe asthma exacerbation, mean (SD) |  | 0.24 (0.80) | 0.28 (0.55) | *0* |
| ER visits in the past 12 months, mean(SD) |  | 0.16 (0.59) | 0.02 (0.20) | 0 |
| Hospital admissions in the past 12 months, mean(SD) |  | 0.10 (0.38) | 0.02 (0.14) | 0 |
| Invasive ventilation, mean(SD) |  | 0.01 (0.10) | 0 (0.04) | 0.01 |
| Percent predicted FEV1, mean(SD) |  | 72.22 (19.75) | 83.48 (19.19) | 0 |
| Reversibility of FEV1, mean(SD) |  | 5.92 (9.42) | 2.48 (5.49) | 0 |
| FEV1/FVC ratio, mean(SD) |  | 0.68 (0.12) | 0.70 (0.11) | 9e-04 |
| IgE count (IU/mL), mean(SD) |  | 425.35 (912.57) | 269.17 (630.26) | 0 |
| FeNO (ppb), mean(SD) |  | 41.97 (32.69) | 40.43 (32.30) | 0.17 |
| Blood eosinophil count (mL), mean(SD) |  | 429.93 (327.83) | 400.48 (313.01) | 0.02 |
| Chronic rhinosinusitis (CRS) and nasal polyps (NP), n(%) | CRS without NP | 1184 (19.74%) | 181 (22.21%) | 1e-04 |
|  | CRS with NP | 1827 (30.46%) | 293 (35.95%) |  |
|  | None | 2988 (49.81%) | 341 (41.84%) |  |
| Eczema, n(%) | Y | 746 (12.44%) | 217 (26.63%) | 0 |
|  | N | 5253 (87.56%) | 598 (73.37%) |  |
| Anxiety, n(%) | Y | 435 (7.25%) | 66 (8.10%) | 0.42 |
|  | N | 5564 (92.75%) | 749 (91.90%) |  |
| Depression, n(%) | Y | 416 (6.93%) | 98 (12.02%) | 0 |
|  | N | 5583 (93.07%) | 717 (87.98%) |  |
| Allergic rhinitis, n(%) | Y | 3780 (63.01%) | 620 (76.07%) | 0 |
|  | N | 2219 (36.99%) | 195 (23.93%) |  |
| Steroid sparing, n(%) | Y | 58 (0.97%) | 48 (5.89%) | 0 |
|  | N | 5941 (99.03%) | 767 (94.11%) |  |
| Pneumonia, n(%) | Y | 550 (9.17%) | 106 (13.01%) | 6e-04 |
|  | N | 5449 (90.83%) | 709 (86.99%) |  |
| Sleep apnea, n(%) | Y | 750 (12.50%) | 183 (22.45%) | 0 |
|  | N | 5249 (87.50%) | 632 (77.55%) |  |
| LAMA, n(%) | Y | 337 (5.62%) | 227 (27.85%) | 0 |
|  | N | 5662 (94.38%) | 588 (72.15%) |  |
| LABA/LAMA, n(%) | Y | 11 (0.18%) | 9 (1.10%) | 2e-04 |
|  | N | 5988 (99.82%) | 806 (98.90%) |  |
| Long-term use of OCS, n(%) | Y | 422 (7.03%) | 92 (11.29%) | 0 |
|  | N | 5577 (92.97%) | 723 (88.71%) |  |

*Note: For continuous predictors, we reported mean (SD), and for categorical predictors, we reported available N (% proportion); p-values are based on the Wilcoxon rank-sum test and chi-square (Fisher’s exact test).

e-Figure 1: Structure plot based on the full set of 28 risk predictors


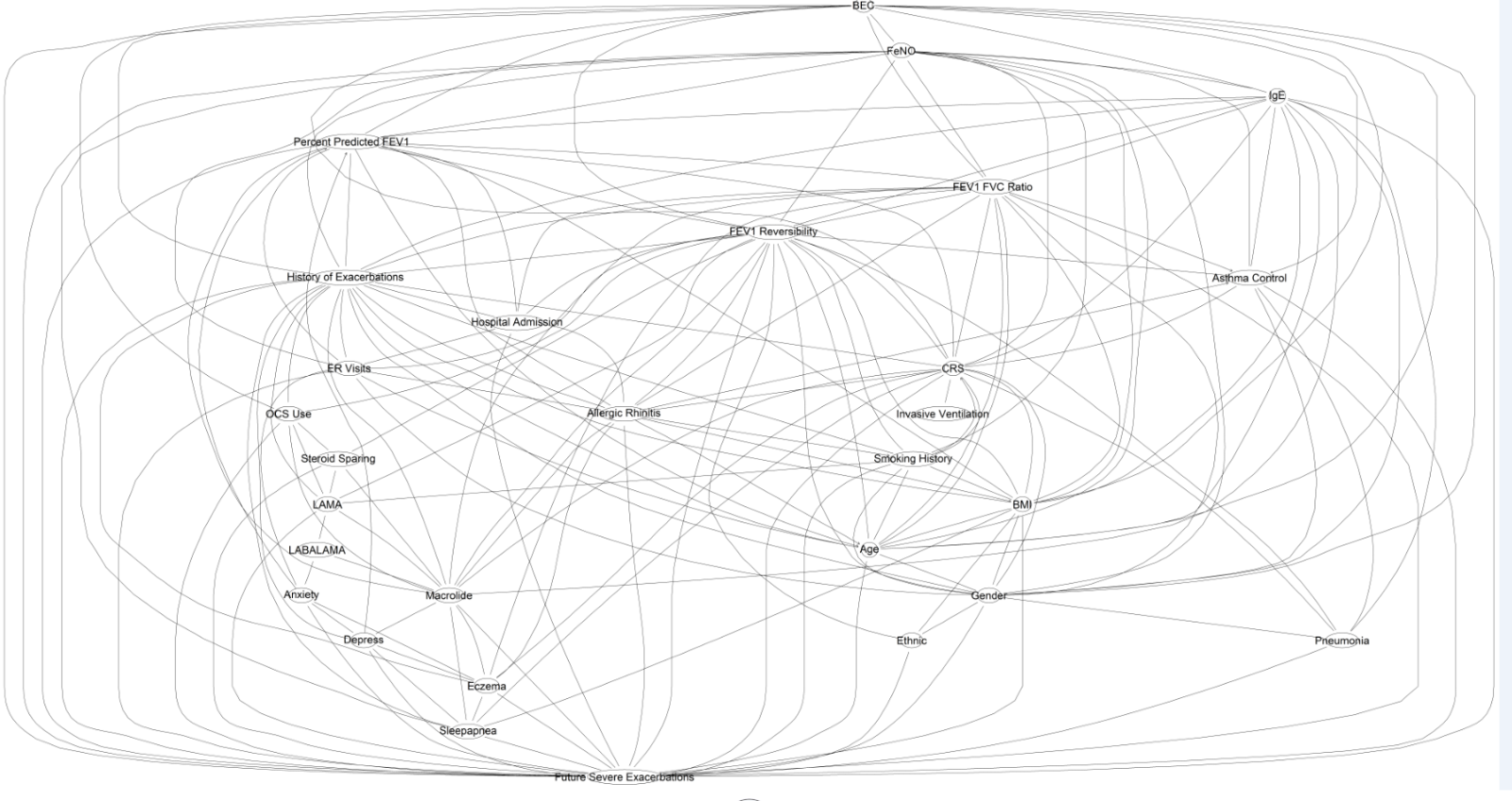


e-Figure 2: Calibration plots using train-test data

1. Risk prediction of  ≥1 severe exacerbations


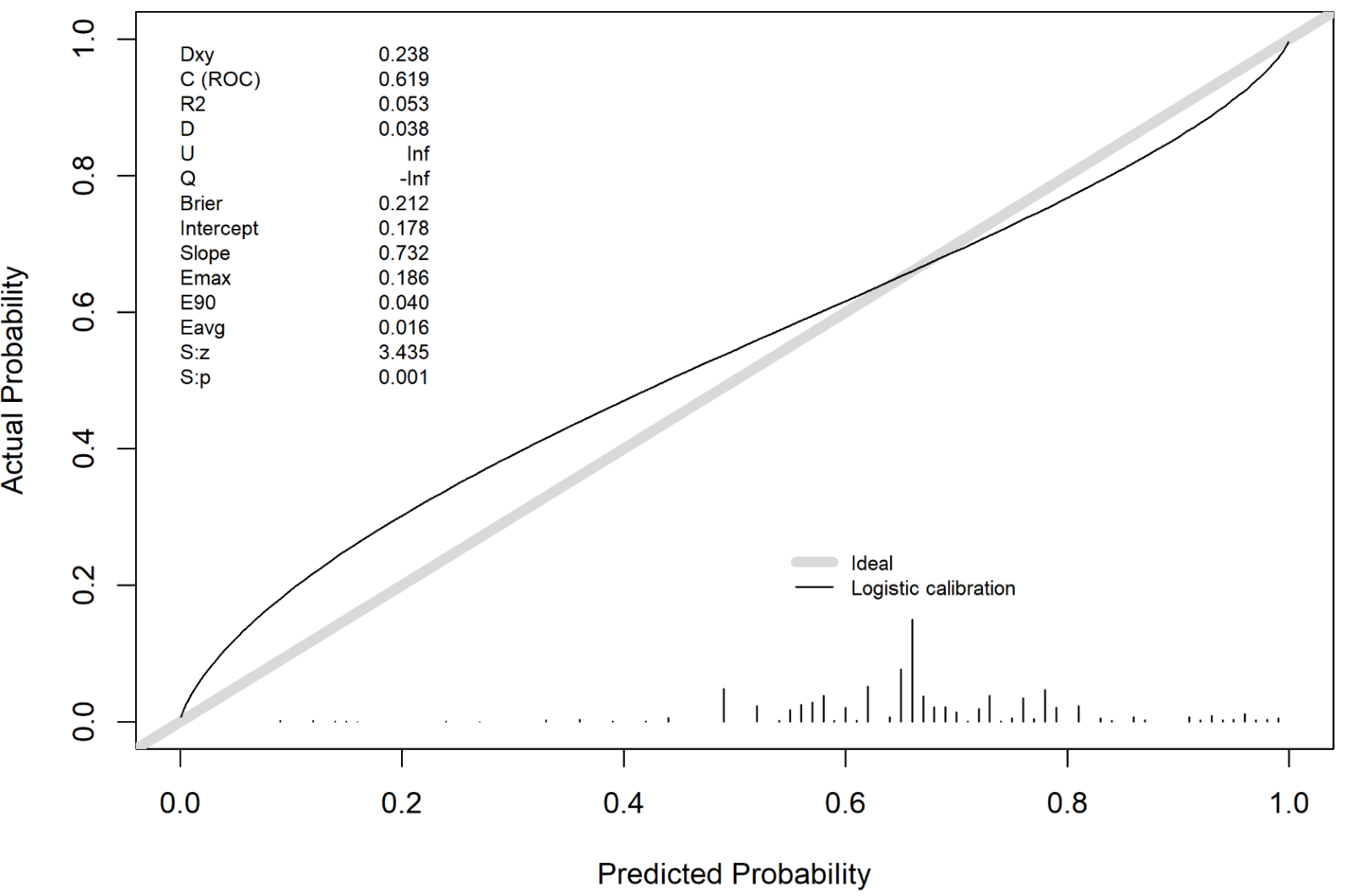


1. Risk prediction of  ≥2 severe exacerbations


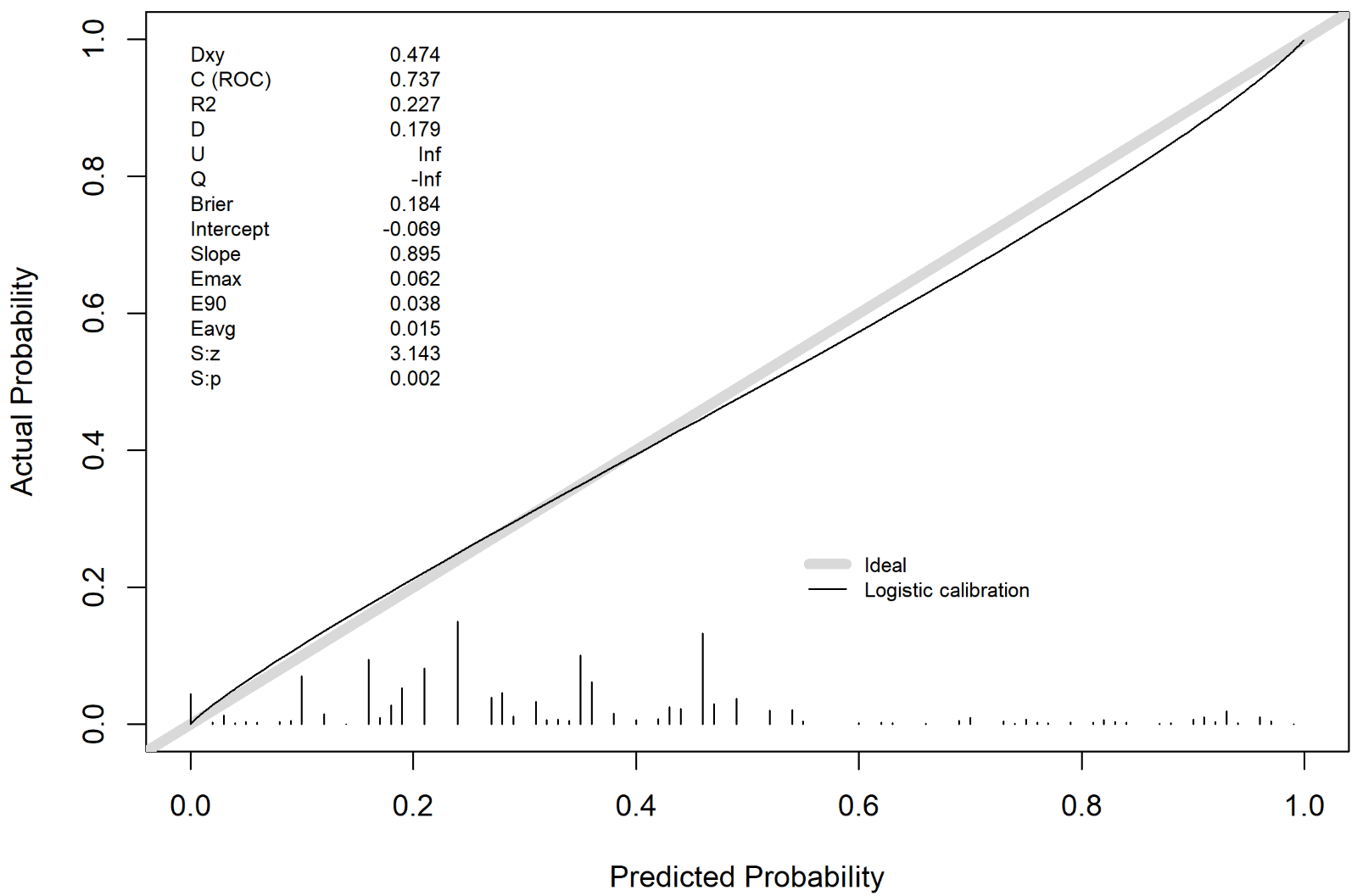


### **10. References**

1. Quanjer, Philip H., Sanja Stanojevic, Tim J. Cole, Xaver Baur, Graham L. Hall, Bruce H. Culver, Paul L. Enright et al. Multi-ethnic reference values for spirometry for the 3–95-yr age range: the global lung function 2012 equations. 2012; 1324-1343.
2. Stekhoven DJ, Bühlmann P. MissForest—non-parametric missing value imputation for mixed-type data. Bioinformatics 2012;28(1):112-118.
3. Hartemink AJ. Principled computational methods for the validation discovery of genetic regulatory networks (Doctoral dissertation, Massachusetts Institute of Technology). Available from: http://hdl.handle.net/1721.1/8699
4. He H, Bai Y, Garcia EA, Li S. ADASYN: Adaptive synthetic sampling approach for imbalanced learning. In 2008 IEEE international joint conference on neural networks (IEEE world congress on computational intelligence) 2008;(pp. 1322-1328).
5. Lee TY, Sadatsafavi M, Yadav CP, Price DB, Beasley R, Janson C, Koh MS, Roy R, Chen W. Individualised risk prediction model for exacerbations in patients with severe asthma: protocol for a multicentre real-world risk modelling study. BMJ open. 2023;13(3):e070459.
